# How Alligator Immune Peptides Kill Gram-Negative Bacteria: A Lipid-Scrambling, Squeezing, and Extracting Mechanism Revealed by Theoretical Simulations

**DOI:** 10.3390/ijms241310962

**Published:** 2023-06-30

**Authors:** Xiangyuan Li, Lei Fu, Shan Zhang, Yipeng Wang, Lianghui Gao

**Affiliations:** 1 Key Laboratory of Theoretical and Computational Photochemistry, Ministry of Education, College of Chemistry, Beijing Normal University, Beijing 100875, China; xiangyuan@mail.bnu.edu.cn (X.L.); 201731150043@mail.bnu.edu.cn (L.F.); shanzhang@mail.bnu.edu.cn (S.Z.); 2 Department of Biopharmaceutical Sciences, College of Pharmaceutical Sciences, Soochow University, Suzhou 215123, China; yipengwang@suda.edu.cn

**Keywords:** antimicrobial peptide, Gram-negative bacteria, coarse-grained, molecular dynamics simulation

## Abstract

Alligator sinensis cathelicidins (As-CATHs) are antimicrobial peptides extracted from alligators that enable alligators to cope with diseases caused by bacterial infections. This study assessed the damaging effects of sequence-truncated and residue-substituted variants of As-CATH4, AS4-1, AS4-5, and AS4-9 (with decreasing charges but increasing hydrophobicity) on the membranes of Gram-negative bacteria at the molecular level by using coarse-grained molecular dynamics simulations. The simulations predicted that all the variants disrupt the structures of the inner membrane of Gram-negative bacteria, with AS4-9 having the highest antibacterial activity that is able to squeeze the membrane and extract lipids from the membrane. However, none of them can disrupt the structure of asymmetric outer membrane of Gram-negative bacteria, which is composed of lipopolysaccharides in the outer leaflet and phospholipids in the inner leaflet. Nonetheless, the adsorption of AS4-9 induces lipid scrambling in the membrane by lowering the free energy of a phospholipid flipping from the inner leaflet up to the outer leaflet. Upon binding onto the lipid-scrambled outer membrane, AS4-9s are predicted to squeeze and extract phospholipids from the membrane, AS4-5s have a weak pull-out effect, and AS4-1s mainly stay free in water without any lipid-extracting function. These findings provide inspiration for the development of potent therapeutic agents targeting bacteria.

## 1. Introduction

Antimicrobial resistance (AMR) poses a significant threat to humans, animals, and plants [1,2]. Based on the trends of increasing drug resistance, the O’Neill report estimates that unless appropriate action is taken, AMR will cause up to 10 million deaths annually by the year 2050 [3]. There is an urgent need to seek new antimicrobial agents or to develop alternatives to conventional antibiotics. Antimicrobial peptides (AMPs), which can be found in most living organisms, are small peptides typically consisting of five to a hundred amino acid residues, including both basic (arginine, lysine, histidine) and hydrophobic residues (often exceeding 50%). They have broad-spectrum antimicrobial and immune-modulatory effects against infectious Gram-positive and Gram-negative bacteria, viruses, and fungi [4,5,6,7]. Therefore, variations of natural AMPs or syntheses of new AMPs are potentially effective antimicrobial agents.

**Figure 1:**
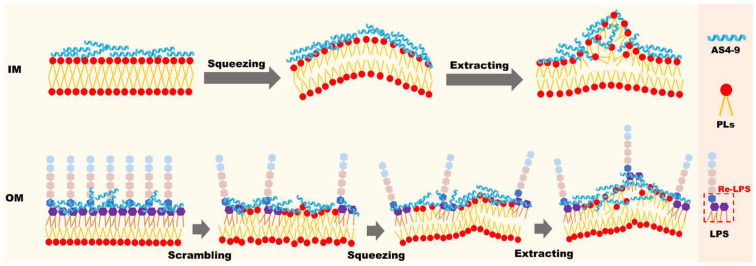
Schematic illustration of the process by which AS4-9 destroys the inner and outer membrane of Gram-negative bacteria.

Crocodiles have much stronger immune systems than humans [8]. Their serum and leukocyte extracts have extensive and broad-spectrum antibacterial effects against pathogens in water, thus allowing their wounds to quickly recover [9,10,11]. In a recent study, Chen et al., identified six novel AMPs from the species *Alligator sinensis*, cathelicidin (As-CATH) 1–6, and found that As-CATHs are pivotal immune modulators for the host immune response to resist microbial infections [12]. Among the six AMPs, As-CATH4, which adopts an α-helix conformation in the N-terminal, has low toxicity and is the most valuable antimicrobial peptide against human diseases. Subsequently, Zhang et al., designed twelve derivatives based on the N-terminal α-helical fragment AS4 with 17 residues of As-CATH4 [13]. Three of these derivatives (AS4-1, AS4-5, and AS4-9) with a variety of electric charges (+9, +7, and +5, respectively) and hydrophobicity (−1.09, −0.07, and 1.08, respectively), were intensively studied. Killing kinetics and bacterial fluorescent dye release experiments have demonstrated that the antibacterial rates of the three derivatives are in the order of AS4-1 < AS4-5 < AS4-9. Our prior study based on molecular dynamics (MD) simulations demonstrated that a good balance between the electric charges and hydrophobicity is an important factor affecting the bactericidal effects of AMPs [13]. In this study, more simulations were performed to study the detailed bactericidal mechanisms of the three derivatives against both the inner membrane (IM) and outer membrane (OM) of Gram-negative bacteria at the molecular level. Unlike “barrel-stave-drilling” [14,15], “carpet” [16,17], and “toroidal wormhole” models that have been previously proposed [18], we observed a novel lipid-scrambling, squeezing, and extracting mechanism, as illustrated in Figure 1. At sufficiently high peptide concentrations, the derivative AS4-9 with the most significant bactericidal activity destroys the integrity of the IM of Gram-negative bacteria by squeezing and extracting phospholipids (PLs) from the proximal leaflet. The lipid-extracting events caused by AS4-5 only occur occasionally, and AS4-1 has almost no ability to extract lipids and perturb the membrane seriously. However, none of the three derivatives directly destroy the integrity of the OM of Gram-negative bacteria, whose outer leaflet is composed of more protective lipopolysaccharide (LPS). Alternatively, the binding of AS4-9 onto the outer leaflet significantly lowers the free energy required for a phospholipid to flip from the inner leaflet up to the outer leaflet and results in a lipid-scrambled membrane. The scrambled and LPS/PL-mixed membrane is more susceptible to AS4-9, and PLs are squeezed and subsequently extracted by the peptides. This novel antimicrobial mechanism provides inspiration for the design of potent therapeutic peptides targeting (especially Gram-negative) bacteria.

## 2. Results

### 2.1. Interactions between AS4 Derivatives and the Inner Membrane Model

**Figure 2:**
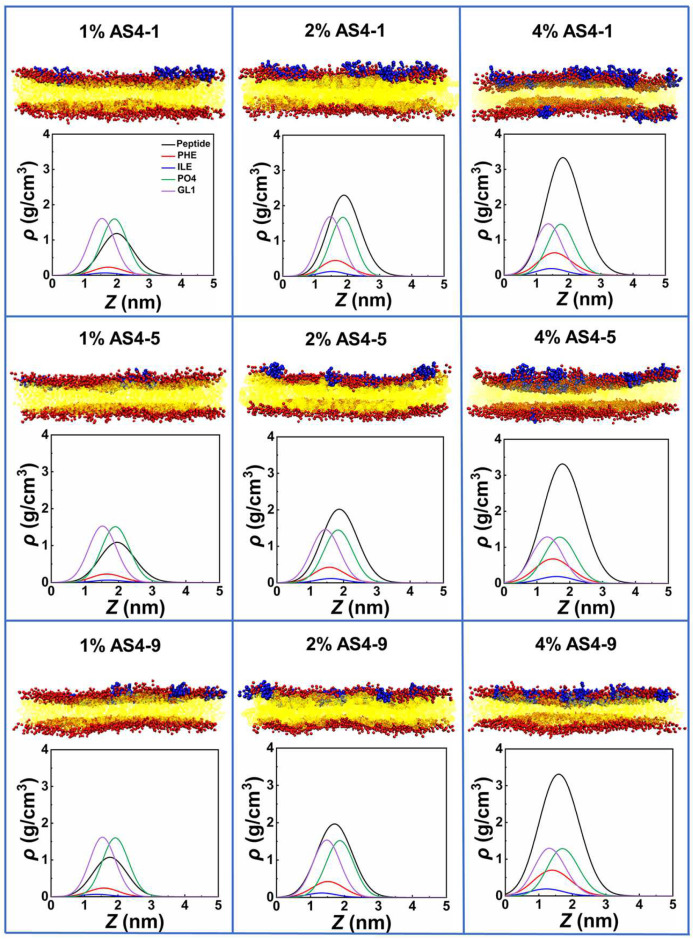
Snapshots of AS4-1, AS4-5, and AS4-9 binding to inner membrane models of Gram-negative bacteria at P/L molar ratios of 1%, 2%, and 4% (upper panel) and the corresponding density distribution profiles of the phosphate and glycerol headgroups of lipids, centers of mass of the peptides, and typical hydrophobic side-chain beads of the peptides along the direction of the bilayer normal (lower panel). The peptides, lipid head groups, and lipid tails are represented by blue, red, and yellow beads, respectively.

**Figure 3:**
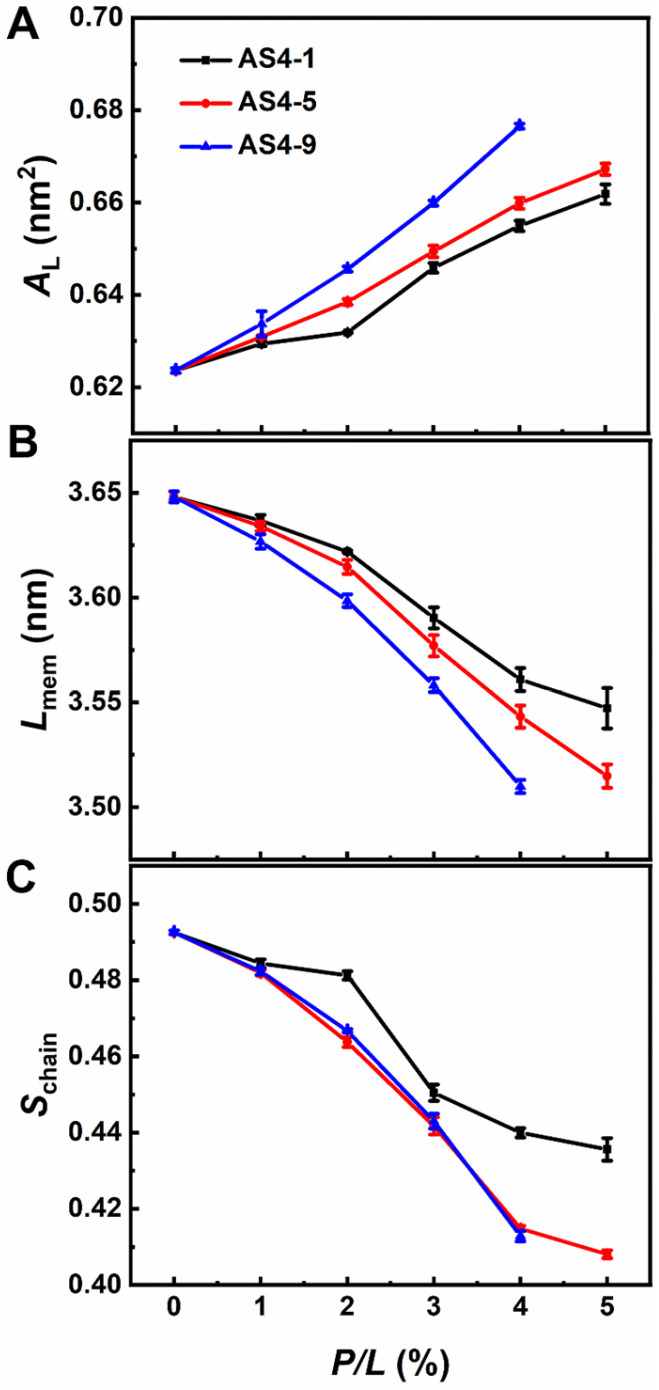
Alterations of area per lipid, *A*_L_ (**A**), membrane thickness, *L*_mem_ (**B**), and lipid tail order, *S*_chain_ (**C**) of IM model induced by AS4-1, AS4-5, and AS4-9 at various peptide concentrations.

We first performed coarse-grained (CG) simulations on the interactions between AS4 derivatives AS4-1, AS4-5, and AS4-9 and inner membrane models at peptide concentrations defined by peptide/lipid (*P*/*L*) molar ratios ranging from 1% to 10%. Herein, the IM was modelled as a symmetrical phospholipid bilayer composed of mixed zwitterionic phosphatidylethanolamine (POPE) and anionic phosphatidylglycerol (POPG) phospholipids with a molar ratio of 3:1. The molecular and CG structures of the lipids and peptides are shown in Figures S1 and S2. At relatively low peptide concentrations (*P*/*L* ≤ 4%), the three peptides bind to the membrane in a similar way. They all adsorb onto the upper leaflet due to electrostatic attraction. The snapshots in Figure 2 show that the membranes are relatively flat, and their integrities are not significantly modified, thus indicating that the peptides are mainly trapped in the lipid head−water interfaces. However, the density distribution profiles of the peptides and lipid head groups show that the penetration depths of the three derivatives are different. For example, the centers of mass (CMs) of AS4-1 and AS-5 are higher than the phosphonate (PO4) beads; however, the CM of AS4-9 is found between the PO4 and glycerol (GL1) beads. In addition, the density distributions of typical hydrophobic side-chain beads of the peptides, such as phenylalanine (PHE) and isoleucine (ILE), further demonstrate that AS4-9 penetrates the membrane more deeply. The structures of the membrane are also subjected to varying degrees of perturbation at these relatively low peptide concentrations. As shown in Figure 3, all three peptides result in increased area per lipid and decreased membrane thickness and lipid orientation order when more peptides bind to the membrane, whereas AS4-9 is the most pronounced.

**Figure 4:**
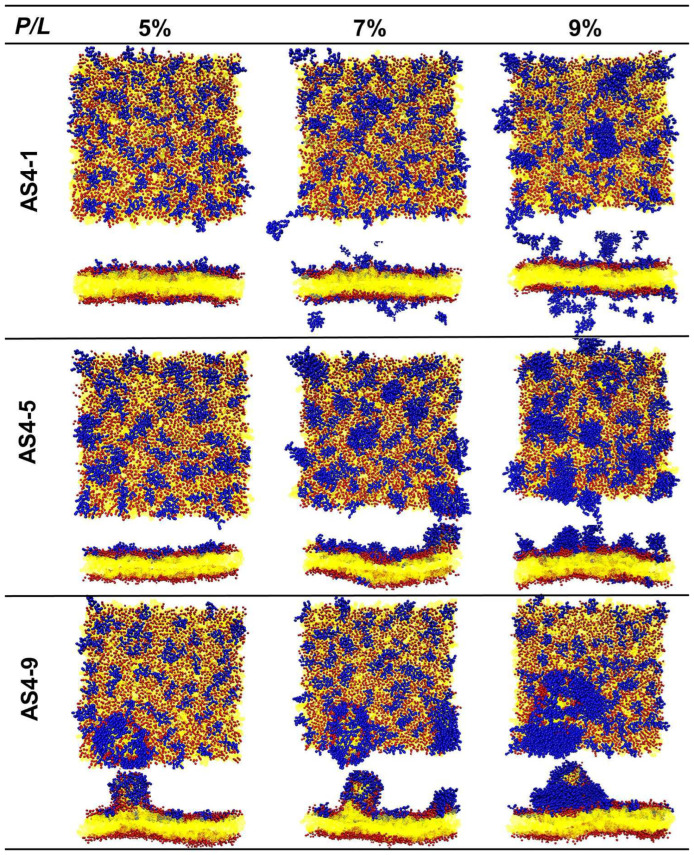
Top and cross-sectional views of snapshots of AS4 derivatives acting on inner membrane of Gram-negative bacteria at relatively high peptide concentrations.

**Figure 5:**
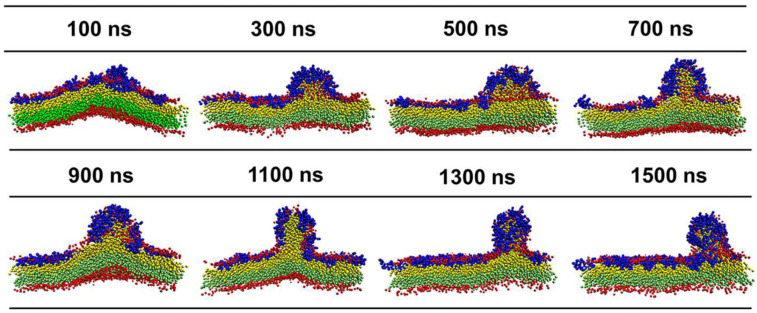
Time sequences of side-view snapshots of the inner membrane model of Gram-negative bacteria treated with AS4-9 at a peptide concentration of *P*/*L* = 5%. To distinguish lipids from the upper and lower leaflets, their tail beads are represented by yellow and green, respectively.

**Figure 6:**
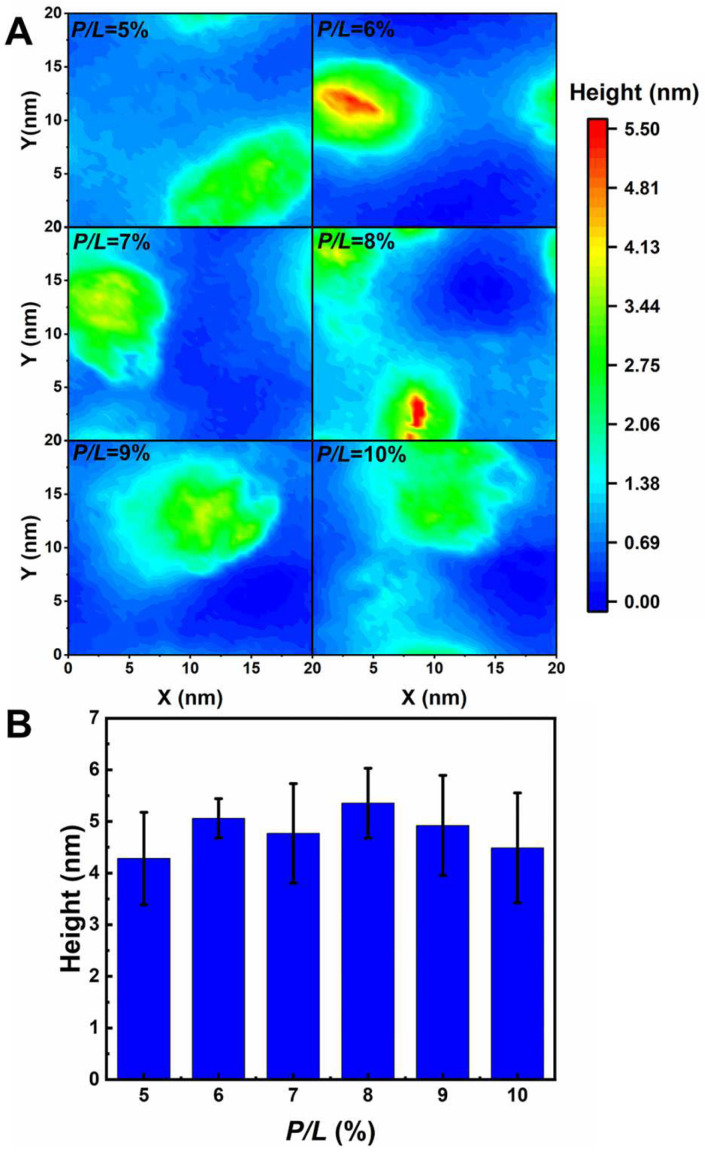
(**A**) Two-dimensional height contours of protrusions induced by AS4-9s on inner membrane models of Gram-negative bacteria. (**B**) The height of protrusions corresponding to (**A**).

When the *P*/*L* molar ratio exceeds 5%, the landscapes of the IM models disrupted by the three peptides are extremely distinguished (Figure 4). At a *P*/*L* of 5%, the negative charges of POPG of the membrane are completely neutralized by the positive charges of AS4-1s. Additional peptides stay free in the solvent rather than binding to the membrane (some peptides may even move to the opposite leaflet due to the periodic boundary conditions). Additionally, the integrity of the membrane remains intact even at very high peptide concentrations. More AS4-5s can be adsorbed onto the membrane because they carry fewer charges. Moreover, their penetration into the membrane squeezes nearby lipids, thus leading to a curved membrane. Tiny buds composed of lipids and peptides nucleated on the membrane surface are occasionally observed. In contrast, the binding of AS4-9s onto the IM models induces noticeable protrusions. The time-sequenced snapshots in Figure 5 show that the binding of AS4-9s first bends the membrane into a curved shape. When more peptides penetrate the membrane, they squeeze the lipids and even extract them out of the mother membrane at locations where the local peptide concentrations are relatively high. The extrusions continuously grow to release the peptide-induced compression. Finally, the membrane recovers to a flat patch with one or multiple extruded buds on the peptide-bound leaflet. The snapshots also show that the lipids in the extrusions mainly originate from the upper leaflet. The heights of the protrusions were estimated by monitoring the positions of PO4 beads in the upper leaflet and are shown in Figure 6A via 2D contours. At 5% ≤ *P*/*L*≤ 10%, the protrusions have similar heights near 5 nm (Figure 6B); however, massive peptides can induce multiple protrusions. Atomic force microscopy topography analysis of the POPC (1-Palmitoyl-2-oleoyl-sn-glycero-3-phosphocholine)/POPG bilayer membrane deposited on a mica surface observed bulges with the same ordered heights as our simulations [13]. Furthermore, almost no lipids flip-flop between the two leaflets. Translocation of the peptide was also not observed.

### 2.2. Interactions between AS4 Derivatives and the Outer Membrane Model

**Figure 7:**
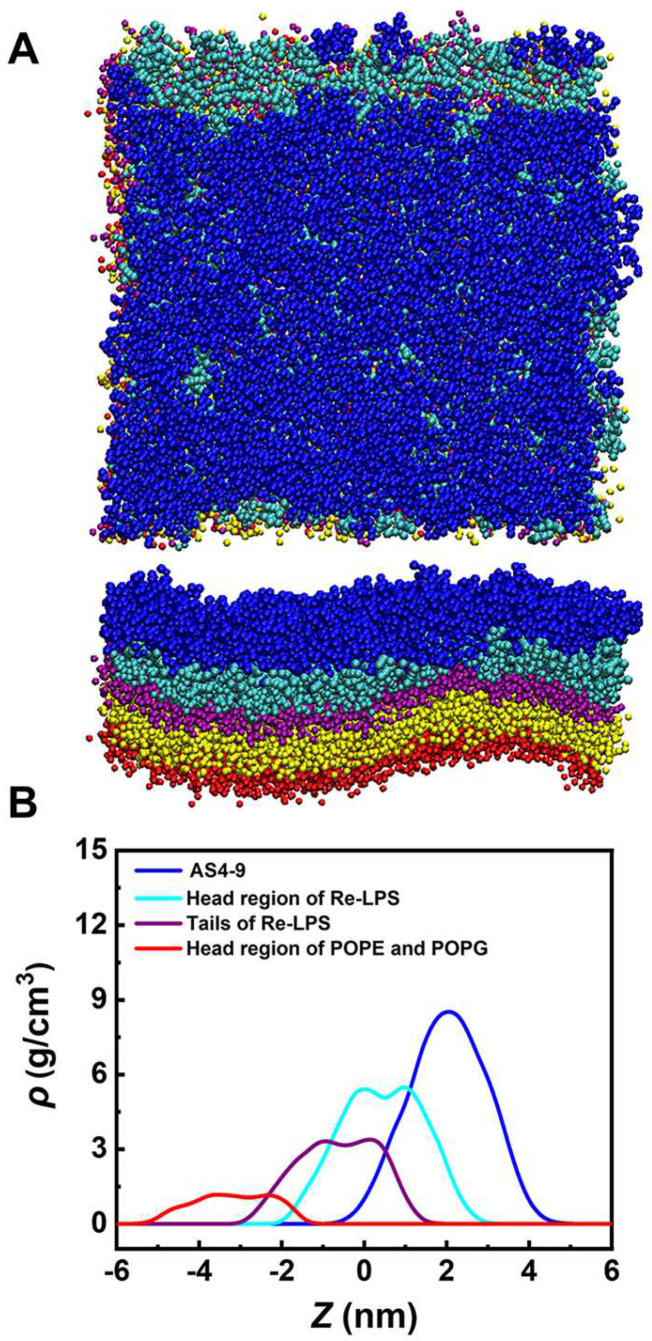
(**A**) Snapshot of AS4-9s interacting with the OM of Gram-negative bacteria at a peptide concentration *P*/*L* of 15%. The hydrophilic head and hydrophobic tail of Re-LPS are represented by cyan and purple beads, respectively. (**B**) Density distribution profiles of Re-LPS and AS4-9 along the direction of the bilayer normal.

**Figure 8:**
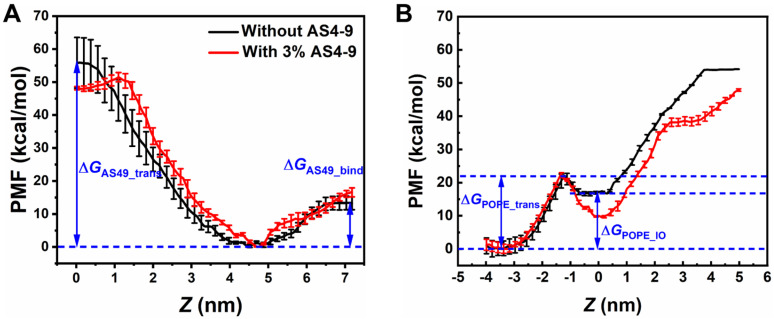
(**A**) Potential of mean force for an AS4-9 molecule penetrating from the Re-LPS leaflet to the hydrophobic center of an asymmetric Gram-negative bacterial outer membrane model. The average position of the terminal tail beads of the phospholipids in the inner leaflet is taken as the reference zero point. (**B**) Potentials of mean force for a POPE flipping from the phospholipid leaflet to the Re-LPS leaflet free of peptides and bound by AS4-9 at a peptide concentration of *P*/*L* = 3%. The average position of the GL1 head beads of the Re-LPS in the upper leaflet is taken as the reference zero point.

To evaluate the interactions between AS4 derivatives and the OM of Gram-negative bacteria, the OM was modelled as an asymmetric bilayer composed of Re-LPS (rough (R)-form LPS isolated and purified from *Escherichia coli*, i.e., lipid A plus two 3-deoxy-D-manno-oct-2-ulosonic acid (KDO) sugars) in one leaflet and POPE/POPG in another leaflet. Figure 7 shows that the OM models treated with the most antibacterial derivative (AS4-9) maintain their intact bilayer structures even at *P*/*L* up to 15%, at which point the negative charges of Re-LPSs are fully neutralized by the peptides. The AMPs only penetrate the sugar groups shallowly. Enhanced umbrella sampling simulations also demonstrated that AS4-9 can hardly translocate across the OM because the energy barrier is as high as 59 kcal/mol (Δ*G*_AS4-9_trans_) (Figure 8A). An increase of the peptide concentration to 3% reduces the energy barrier to 48 kcal/mol but is still not low enough to allow the peptide to cross the OM. These observations seem to conflict with our previous in vitro experiments, in which AS4-9 exhibited potent antimicrobial activities against Gram-negative bacteria by inducing massive cell collapse, surface fibrosis, and cellular inclusion leakage [13]. Hence, it is possible that there exists an undisclosed antimicrobial mechanism yet to be elucidated by the simulations.

Our recent study on another type of AMP known as polymyxin B (PmB) found that PmBs also do not directly disrupt the integrity of the OM. Alternatively, their binding loosens the packing of Re-LPS and induces unbalanced bending torque between the inner and outer leaflets, which correspondingly triggers phospholipids to flip from the inner leaflet to the outer leaflet to compensate for the stress deformation. The lipid homeostasis and asymmetry of the OM are consequently altered, and the membrane becomes less protective [19]. This result inspired us to speculate that AS4 derivatives may perturb the homeostasis of the OM in a similar way. To test this hypothesis, we calculated the potential of mean force (PMF) of pulling a POPE molecule from the lower leaflet composed of phospholipids to the upper leaflet composed of Re-LPS before and after treatment with AS4-9. As shown in Figure 8B, a lipid requires 23 kcal/mol energy (Δ*G*_POPE_trans_) to penetrate the hydrophobic tail region of a peptide-free bilayer. However, rather than flipping up to the upper leaflet, the lipid still favors dwelling in the inner leaflet because the energy difference between the two states (Δ*G*_POPE_IO_) is up to 19 kcal/mol. If the Re-LPS leaflet is bound by a certain amount of AS4-9s (such as *P*/*L* = 3%), the energy required for a POPE in the inner leaflet to enter the hydrophobic tail of the bilayer slightly decreases to 22 kcal/mol. Surprisingly, the energy difference for the lipid to dwell in the two leaflets is significantly lowered to 9.5 kcal/mol. This effect implies that the POPE molecule more likely flips up to the upper Re-LPS leaflet, thus leading to a scrambled OM bilayer.

**Figure 9:**
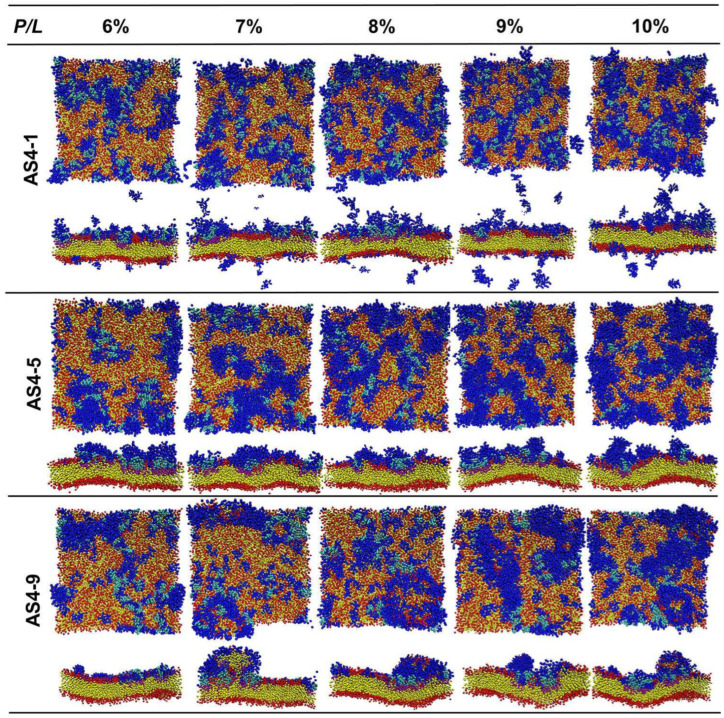
Snapshots of AS4s binding to scrambled OM of Gram-negative bacteria with deficient Re-LPS (*r_LPS_* = 10%).

We next studied the actions of AS4 derivatives on the scrambled OM models of Gram-negative bacteria. Herein, a lipid-scrambled OM (s-OM) was modelled as an asymmetric bilayer with one leaflet composed of mixed Re-LPS/POPE/POPG and the other leaflet composed of POPE/POPG as an ordinary OM model. The molar proportion of Re-LPS in the outer leaflet (*r_LPS_*) was set to 75%, 50%, 25%, and 10% to mimic different deficiencies of LPS. The molar ratios between POPE and POPG in both leaflets were the same and equal to those in the ordinary OM model. Figure S3 shows that AS4-9 favors binding to Re-LPSs over PLs. With a similar action as with an ordinary OM, if the scrambled OM comprises sufficient Re-LPS (*r_LPS_* ≥ 50), the bilayer structure of the s-OM is not significantly modified by AS4-9s (even at peptide concentrations *P*/*L* as high as 9%). On an s-OM with *r_LPS_* = 25%, AS4-9s can bind to both Re-LPSs and PLs at peptide concentrations *P*/*L* > 7%. Subsequently, the bilayer becomes more curved, and few lipids are observed to be extracted from the membrane. In an s-OM model with more deficient LPSs (*r_LPS_* = 10%), as in the IM model, the binding of AS4-9s induces protrusion budding at *P*/*L* > 6% (Figure 9). Composition analysis shows that the percentage of POPE, POPG, and Re-LPS in the budding is approximately the same as the ratio of lipids in the outer leaflet (Re-LPS: POPE: POPG = 1:8:1) (Figure S4). The morphologies of s-OMs with deficient LPSs (*r_LPS_* = 10%) treated with AS4-1s and AS4-5s are also shown in Figure 9. As in the case of the IM, fewer AS4-1s can bind to the membrane and do not significantly perturb the bilayer structure. AS4-5s are shallowly adsorbed on the s-OMs and randomly induce tiny lipid extrusions. These results suggest that the induction of lipid-scrambling is the first step for AS4 derivatives to exert antimicrobial effects.

## 3. Discussion

The design of new antimicrobial agents based on natural AMPs is a potent strategy to cope with drug-resistance threats caused by the abuse of conventional antibiotics. Naturally occurring peptides As-CATHs from the Chinese *Alligator sinensis* provide good templates [13]. A series of peptide derivatives with different hydrophobicity and positive charge were designed based on AS4, the N-terminal α-helical fragments of As-CATH4. It has been shown that they have various antibacterial effects. Considering three derivatives (AS4-1, AS4-5, and AS4-9) as examples, this work investigated their antimicrobial mechanisms against both the IM and OM of Gram-negative bacteria at the molecular level in detail by using molecular dynamics simulations. A novel killing mechanism was found that provides inspiration for the development of potent therapeutic agents.

Electrostatic interactions are the main driving forces for cationic AMPs to target anionic bacterial membranes. However, our studies on AS4 derivatives show that the most highly charged peptide (AS4-1) exerts the weakest bactericidal effect. A small amount of AS4-1 binding to the membrane can effectively neutralize the charges on the membrane. The fast adsorption saturation results in ineffective contact between the peptide and lipids. Moreover, the fewer hydrophobic residues of AS4-1 also reduce its ability to perturb membrane integrity. In contrast, AS4-9 has four fewer positive charges than AS4-1, thus almost double the amount of AS4-9 can consequently be adsorbed onto the membrane and interact with lipids. The larger hydrophobic portion of AS4-9 also permits it to penetrate the membrane more deeply and disrupt the packing of lipids. Nonetheless, continuing to increase hydrophobicity and decrease charges correspondingly reduces the antimicrobial activity of the peptide. For example, AS4-11 and AS4-12 did not exhibit detectable antimicrobial activity [13]. Peptides with extremely low positive charges and high hydrophobicity may associate into clusters in solvent, thus leading to ineffective contacts and interactions between peptides and lipids. These results indicate that moderate charges and hydrophobicity are essential to achieve optimal antimicrobial activity.

AMPs display a broad spectrum of antibacterial activity against Gram-negative and Gram-positive bacteria with a low propensity to induce drug resistance because they mainly target cell membranes rather than proteins or nucleic acids [20]. A series of models have proposed that AMPs tend to insert into the lipid head groups and disturb the membrane via the formation of membrane pores or membrane morphology changes, such as “toroidal”, “barrel-stave”, and “carpet” models [14,15,16,17,18]. In these models, peptides insert into the membrane (forming water-permeable pores) or dissolve the membrane into micelles. However, we found that AS4 derivatives exert antibacterial action in a different manner. The penetration of hydrophobic residues into the membrane disturbs the packing of lipids and induces unbalanced tension between the two leaflets. If the hydrophobic portions of the peptides are large and penetrate the lipid tail region deeply, they may alter the packing of lipids in both leaflets; specifically, lipids in the proximal leaflet are compressed, and lipids in the distal leaflet are stretched. Subsequently, the bilayer will become curved. To release the unbalanced tension, peptides may insert perpendicularly into the membrane, thus forming transmembrane (like barrel-stave) pores and allowing for the membrane to maintain a symmetric and equilibrium bilayer structure. In addition, both peptides and lipids in the proximal leaflet can move to the distal leaflet through a toroidal-like pore or carpet collapsing. Our previous study found that AMPs with lengths comparable to the membrane thickness and similar amphiphilic portions, such as Magainin, Melittin, Tachyplesin, and Protegrin, employ this killing mechanism [21,22]. Unfortunately, this type of AMP is usually cytotoxic. Alternatively, if the hydrophobic portion of the peptide is relatively small and penetrates the lipid head region shallowly, it can only squeeze the packing of lipids in the proximal leaflet but leaves the distal leaflet almost unperturbed. To release the compression in the proximal leaflet, the protrusion of lipids accompanied by peptides is an effective method. Gomesin and Temporin were observed to destroy the membrane via this mechanism [23,24]. AS4 derivatives comprise only 17 residues, and they (especially AS4-9) also squeeze and extract lipids, as was observed in this study. The intact inner leaflet also suggests that this type of AMP may have less cytotoxicity. All-atom (AA) MD simulations on anisaxins (a kind of helical antimicrobial peptides from marine parasites) interacting with phospholipid model membranes also found that AMPs can kill resistant bacteria by lipid extraction and membrane disruption [25]. Both the CG and AA MD simulations suggest that extracting lipids from membrane is an alternative bactericide mechanism in addition to pore formation mechanism.

Symmetric bilayers composed of zwitterionic and anionic phospholipids are commonly selected as membrane models to study the interaction between AMPs and bacterial cells in simulations. However, such models are only valid for mimicking Gram-positive bacterial membranes. Few studies in the literature have investigated the actions of AMPs on the membranes (especially the outer membrane) of Gram-negative bacteria. The manner in which AMPs interact with the OM of Gram-negative bacteria at the molecular level is still a mystery. In this study, we constructed an asymmetric bilayer with one leaflet comprising Re-LPS and another leaflet comprising mixed POPE/POPG lipids as the OM model. Unexpectedly, our simulations showed that the binding of any derivatives of AS4 peptide onto the Re-LPS leaflet cannot directly disrupt the OM structure in the same way as they act on the inner membrane model; however, they do have bactericidal activities against Gram-negative bacteria in wet experiments. This contradiction prompts us to realize that all of the simulations treat bacterial membranes as static models, but cell membranes are homeostatic in vivo [26,27]. Lipid transferases and flippases work together to maintain the lipid asymmetry and barrier function of the OM [28,29,30,31]. We speculate that the disruption of lipid homeostasis might be a possible cell-death pathway caused by AMPs. Our calculations of the potential of mean force for pulling a phospholipid molecule to flip from the inner leaflet to the outer leaflet confirmed this speculation. We found that the binding of AS4-9 significantly lowers the energy difference for the lipid to dwell in the two leaflets. Lipids have a high possibility of flipping up to the Re-LPS leaflet if they can overcome the transmembrane energy barrier [32]. This scenario may become more feasible under the action of transferases and flippases [33,34]. As a result, the homeostasis of the Gram-negative bacteria membrane may be disrupted, and the OM will become lipid-scrambled and less asymmetric. When additional AS4-9 peptides bind to the lipid-scrambled OM model, we found that they squeeze the PLs and even extract the PLs from the membrane. Surface fibrosis observed in *V. parahaemolyticus* treated with AS4 derivatives might be caused by this mechanism [13]. These novel results also suggest that inducing lipid scrambling may be the first and essential step adopted by many types of AMPs to destroy Gram-negative bacteria.

Liposome leakage and cellular inclusion leakage induced by the AS4 derivatives were observed in vivo [13]; however, our simulations did not observe permeable membrane pores in either the IM or OM models. One possible reason is the periodic boundary condition that was used in the simulations, by which the size of the membrane is infinite. If the membrane is a finite-sized patch, the lipid-peptide protrusions may bud off from the mother membrane, thus leaving leakage holes.

## 4. Materials and Methods

### 4.1. Coarse-Grained Molecular Dynamics Simulations

The initial all-atom structures of the three peptides (AS4-1: RGLFKKLLRKIKKGFKK, AS4-5: LGLFKKLLRLIKKGFKK, and AS4-9: LGLFKKLLRLILKGFKL) were constructed by using homology modelling software (UCSF Chimera 1.14 package) [35]. The main difference between the three derivatives lies in the amino acid residues 1, 10, 12, and 18, such that the charge decreases and the hydrophobicity increases in order of AS4-1, AS4-5, and AS4-9. Then the monomer of the peptide was placed on the surface of a pre-equilibrated symmetric membrane composed of 288 mixed POPE and POPG phospholipids (POPE:POPG = 3:1). The system was solvated by 16,635 water molecules and neutralized by 63 Na^+^ ions for the AS4-9 system (or 16,646 water molecules and 65 Na^+^ ions for the AS4-5 system, and 16,648 water molecules and 67 Na^+^ ions for the AS4-9 system) in a box with a size of 8 × 8 × 12 nm^3^. Energy minimization followed by 120 ns all-atom molecular dynamics simulations at a unified temperature of 312 K and constant pressure of 1 bar (NPT ensemble) were performed to obtain stable structures of the peptides at the membrane surfaces. Herein, the GROMOS 53A6 force field [36] in version 5.0.4 of the GROMACS package was employed [37,38,39,40]. The temperature was coupled to a Nose-Hoover heat bath (time constant of 1 ps) [41], and the semi-isotropic pressure was coupled with the Parrinello−Rahman barostat [42,43,44,45] (time constant of 2 ps). The neighbor list was updated every 5 time-steps. Finally, the equilibrated all-atom structures of the three peptides were mapped to coarse-grained structures (Figure S1) by using *martinize.py* in the Martini mapping scheme.

The interactions between peptides and the membrane were evaluated by using coarse-grained molecular dynamics simulations via the package GROMACS (version 5.0.4) with the Martini v2.2 force field [46,47,48,49]. The IM of Gram-negative bacteria was modelled as a symmetrical phospholipid bilayer composed of POPE and POPG (see their molecular and CG structures in Figure S2 and Table S1) with a molar ratio of 3:1. The choice of this molar ratio was based on the results of Carey [50]. Herein, a bilayer composed of 1352 lipids was constructed and solvated in a box of 20.5 × 20.5 × 12 nm^3^ by using *insane.py*. The membrane was neutralized by adding 338 Na^+^ counterions and equilibrated for 1 μs in the NPT ensemble. Afterwards, peptides with *P*/*L* molar ratios ranging from 1% to10% were placed on the surface of the membrane obtained from the last frame of the trajectory. These *P*/*L* molar ratio ranges were selected according to the dynamic giant unilamellar vesicle leakage assay experiments, where the lipid concentration was around 120 μg/mL or 0.17 μmol/mL [13]. Considering that the minimum inhibitory concentrations (MICs) of AS4-9 (with mass of 2286 g/mol) on a couple of bacteria were from 1 to 10 μg/mL (or 0.44 to 4.4 nmol/mL), the P/L molar ratios were set from 1% to 10% in the simulations. Then the normal direction of the simulation box was increased to 20 nm and refilled by water and ions. In this scenario, Cl^-^ ions were used to neutralize the positive charges of peptides, and Na^+^ ions were used to neutralize the negative charges of POPG. After effective energy minimization, simulations in the NPT ensemble were performed for 1.5 μs.

The OM of Gram-negative bacteria is asymmetric, and its outer leaflet exclusively contains lipopolysaccharide molecules, whereas the inner leaflet contains phospholipids in composition similar to those of the inner membrane [2,51,52]. Herein, a bilayer composed of 231 Re-LPSs (see its molecular and CG structures in Figure S2 and Table S1) in one leaflet and 562 POPE and 62 POPG lipids in another leaflet was constructed by using *insane.py* [53] to model the OM [54,55,56]. We have shown that an asymmetric bilayer with such a component ratio is dynamically flat with a matched area between the two leaflets [13,19]. The OM model was subsequently neutralized with Ca^2+^ ions and Na^+^ ions, for which calcium neutralized the charges of Re-LPSs and sodium neutralized the charges of POPG lipids. We also constructed a scrambled OM model with mixed Re-LPSs and PLs in the outer leaflet (the molar ratios of Re-LPS were 75%, 50%, 25%, and 10%). The membranes were equilibrated for 1.5 μs and used for preparing peptide-OM complexes in the same way as for the peptide-IM membrane. Given that Re-LPS has six hydrophobic tails, we used the total number of phospholipids and threefold that of Re-LPS to define the *P*/*L* ratio for systems involving the OM. Finally, simulations up to 3 μs were run in the NPT ensemble for each system.

### 4.2. Potentials of Mean Force for Peptide Translocation and Lipid Flip-Flop

Umbrella sampling and the weighted histogram analysis method (WHAM) were used to calculate the PMF of pulling an AS4-9 or POPE molecule across a bilayer membrane [57]. To calculate the PMF for AS4-9 to penetrate the hydrophobic core of a membrane, we placed an AS4-9 molecule in the solvent at approximately 5 nm from the center of the bilayer. Afterwards, 40 configuration windows were generated at intervals of 0.2 nm along the direction of the membrane normal, wherein the center of mass of the peptide was constrained by a constant harmonic force of 1000 kJ/mol/nm^2^. To calculate the PMF for a POPE molecule flipping from the inner leaflet up to the outer leaflet of the OM model, we randomly chose a POPE molecule in the inner leaflet of a well-equilibrated membrane and generated a series of initial conformations by performing a steered MD. Subsequently, 36 configurations with an interval of 0.2 nm were screened, in which the center of mass of two headgroups (NH3 and PO4 groups) was constrained by a constant harmonic force of 1000 kJ/mol/nm^2^. After pre-equilibration, each window was simulated for 300–800 ns at 312 K to ensure that root-mean-square deviation approached equilibrium (Figure S5), and the trajectory in the last 100 ns was utilized to produce PMF profiles by using the *g_wham* GROMACS tool. Statistical errors were estimated by separating the trajectory in the last 100 ns into three segments.

### 4.3. Analyses

**Orientation order of lipid tails.** The interactions between AS4s and the membrane can change the status of lipids in many aspects. One aspect involves the orientation order of the lipid tails quantifying their mechanical distortions, which was defined as

$$S_{tail}=\frac{1}{2}\left(3\langle {cos}^{2}\varphi \rangle -1\right)$$

Herein, φ is the angle between the normal direction of the membrane and the direction of the hydrocarbon tail. The ensemble average encompassed all of the lipids in the membrane and trajectory samples. The values of −0.5, 0, and 1 imply complete antialignment, random orientation, and alignment, respectively.

**Density distribution profiles.** To obtain the density distribution profiles of different components in the system, the simulation box was divided into slices along the normal direction of the membrane. Then the mass densities of certain groups in each layer were calculated.

**Membrane thickness.** The membrane thickness of a bilayer was calculated by using a grid method to reduce the effect of large membrane fluctuations. Herein, the bilayer was divided into 10 × 10 small cells. In each cell, the local membrane thickness was estimated as the distance between the location of PO4 beads in the upper leaflet and the opposite lower leaflet. The membrane thickness was the average over all the cells.

**Membrane area.** For a relatively flat membrane, the area per lipid was roughly estimated as the projected area of the membrane onto the x-y plane divided by the number of lipids in each leaflet.

**Heights of lipid buddings.** The adsorption of AMPs onto a membrane extracted lipids and induced budding-like extrusions. To quantify the heights of the lipid buddings, the membrane (x-y) plane was divided into grids. The average normal position of PO4 beads from lipids belonging to the AMP-bound leaflet in each cell was estimated and mapped into a two-dimensional grid-height contour.

## 5. Conclusions

In this work, we systematically studied three antimicrobial peptide derivatives, AS4-1, AS4-5, and AS4-9, with different charges and hydrophobicity based on the N-terminal α-helical fragment of one kind of cathelicidin extracted from Chinese *Alligator sinensis* using coarse-grained molecular dynamics simulations. We found that the derivative AS4-9 having moderate charges and hydrophobicity exhibits optimal bactericidal activity. Moderate charges allow the AMPs to quickly target bacterial membrane but not saturate at low concentration. Moderate hydrophobicity enables the AMPs to significantly perturb the membrane structure but not aggregate in solvent. The binding of these short AMPs, especially AS4-9, onto the IM model of Gram-negative bacteria squeezes the lipids in the proximal leaflet and even extracts them out of the membrane at sufficiently high peptide concentration. In contrast, the AMPs cannot directly destroy the structure of the OM model of Gram-negative bacteria. Alternatively, the binding of AS4-9s promotes phospholipids to flip from the inner leaflet to the outer leaflet changing the asymmetry of the OM. Once the OM becomes lipid-scrambled, AS4-9s can bind to phospholipids and extract them out the membrane like they act on IM model. This novel peptide-induced lipid-scrambling, squeezing, and extracting pathway enriches our understanding of antimicrobial mechanisms.

## Data Availability

Authors are willing to provide the data related to this article upon receiving reasonable requests.

## Supplementary Materials

The supporting information can be downloaded at: https://www.mdpi.com/article/10.3390/ijms241310962/s1.
