# Supplementary material for: How Alligator Immune Peptides Kill Gram-Negative Bacteria: A Lipid-Scrambling, Squeezing, and Extracting Mechanism Revealed by Theoretical Simulations
Source: Int J Mol Sci. 2023 Jun 30;24(13):10962. doi: 10.3390/ijms241310962 (PMC10342063; doi:10.3390/ijms241310962)
Supplement: Supplementary file 1 [file ijms-24-10962-s001.zip › ijms-2437098-supplementary.pdf]

International Journal of
Molecular Sciences

MDPI

Supporting Information for

How alligator immune peptides kill Gram-negative bacteria: A lipid-scrambling, squeezing, and extracting mechanism revealed by theoretical simulations

Xiangyuan Li [1,#], Lei Fu [1,#], Shan Zhang[1], Yipeng Wang[2], and Lianghui Gao[1,*]

This file includes:

Table S1

Figure S1 to S5

Table S1. Coarse-grained bead types of POPE, POPG, Re-LPS, AS4-1, AS4-5, and AS4-9 corresponding to the coarse-grained mapping scheme in Figure S1 and Figure S2.

POPE		POPG		Re-LPS					
number	type	number	type	number	type	number	type	number	type
1	Qd	1	P4	1	Qa	15	C1	29	Na
2	Qa	2	Qa	2	P2	16	C1	30	Nda
3	Na	3	Na	3	Nda	17	Na	31	C1
4	Na	4	Na	4	P1	18	Na	32	C1
5	C1	5	C1	5	P2	19	C1	33	Qa
6	C3	6	C3	6	P1	20	C1	34	SC1
7	C1	7	C1	7	Nda	21	C1	35	P2
8	C1	8	C1	8	Qa	22	C1	36	SN0
9	C1	9	C1	9	Na	23	C1	37	P4
10	C1	10	C1	10	Na	24	C1	38	SC1
11	C1	11	C1	11	C1	25	Na	39	P2
12	C1	12	C1	12	C1	26	Nda	40	SN0
				13	C1	27	C1	41	P4
				14	C1	28	C1	42	Qa

AS4-1			AS4-5			AS4-9		
number	type	residue	number	type	residue	number	type	residue
1	Qd	ARG	1	Qd	LEU	1	Qd	LEU
2	N0	ARG	2	AC1	LEU	2	AC1	LEU
3	Qd	ARG	3	P5	GLY	3	P5	GLY
4	P5	GLY	4	P5	LEU	4	Nd	LEU
5	P5	LEU	5	AC1	LEU	5	AC1	LEU
6	AC1	LEU	6	P5	PHE	6	Nda	PHE
7	P5	PHE	7	SC5	PHE	7	SC5	PHE
8	SC5	PHE	8	SC5	PHE	8	SC5	PHE
9	SC5	PHE	9	SC5	PHE	9	SC5	PHE
10	SC5	PHE	10	P5	LYS	10	Nda	LYS
11	P5	LYS	11	C3	LYS	11	C3	LYS
12	C3	LYS	12	Qd	LYS	12	Qd	LYS
13	Qd	LYS	13	P5	LYS	13	Nda	LYS
14	P5	LYS	14	C3	LYS	14	C3	LYS
15	C3	LYS	15	Qd	LYS	15	Qd	LYS
16	Qd	LYS	16	Nd	LEU	16	Na	LEU
17	P5	LEU	17	AC1	LEU	17	AC1	LEU

18	AC1	LEU	18	Nd	LEU	18	P5	LEU
19	Nda	LEU	19	AC1	LEU	19	AC1	LEU
20	AC1	LEU	20	Nd	ARG	20	P5	ARG
21	Nda	ARG	21	N0	ARG	21	N0	ARG
22	N0	ARG	22	Qd	ARG	22	Qd	ARG
23	Qd	ARG	23	Nd	LEU	23	Nd	LEU
24	Nda	LYS	24	AC1	LEU	24	AC1	LEU
25	C3	LYS	25	N0	ILE	25	Nd	ILE
26	Qd	LYS	26	AC1	ILE	26	AC1	ILE
27	Nda	ILE	27	Na	LYS	27	Nda	LEU
28	AC1	ILE	28	C3	LYS	28	AC1	LEU
29	Nda	LYS	29	Qd	LYS	29	Nda	LYS
30	C3	LYS	30	Na	LYS	30	C3	LYS
31	Qd	LYS	31	C3	LYS	31	Qd	LYS
32	P5	LYS	32	Qd	LYS	32	Na	GLY
33	C3	LYS	33	Na	GLY	33	Na	PHE
34	Qd	LYS	34	Na	PHE	34	SC5	PHE
35	P5	GLY	35	SC5	PHE	35	SC5	PHE
36	P5	PHE	36	SC5	PHE	36	SC5	PHE
37	SC5	PHE	37	SC5	PHE	37	P5	LYS
38	SC5	PHE	38	P5	LYS	38	C3	LYS
39	SC5	PHE	39	C3	LYS	39	Qd	LYS
40	P5	LYS	40	Qd	LYS	40	Qa	LEU
41	C3	LYS	41	Qa	LYS	41	AC1	LEU
42	Qd	LYS	42	C3	LYS			
43	Qa	LYS	43	Qd	LYS			
44	C3	LYS						
45	Qd	LYS						

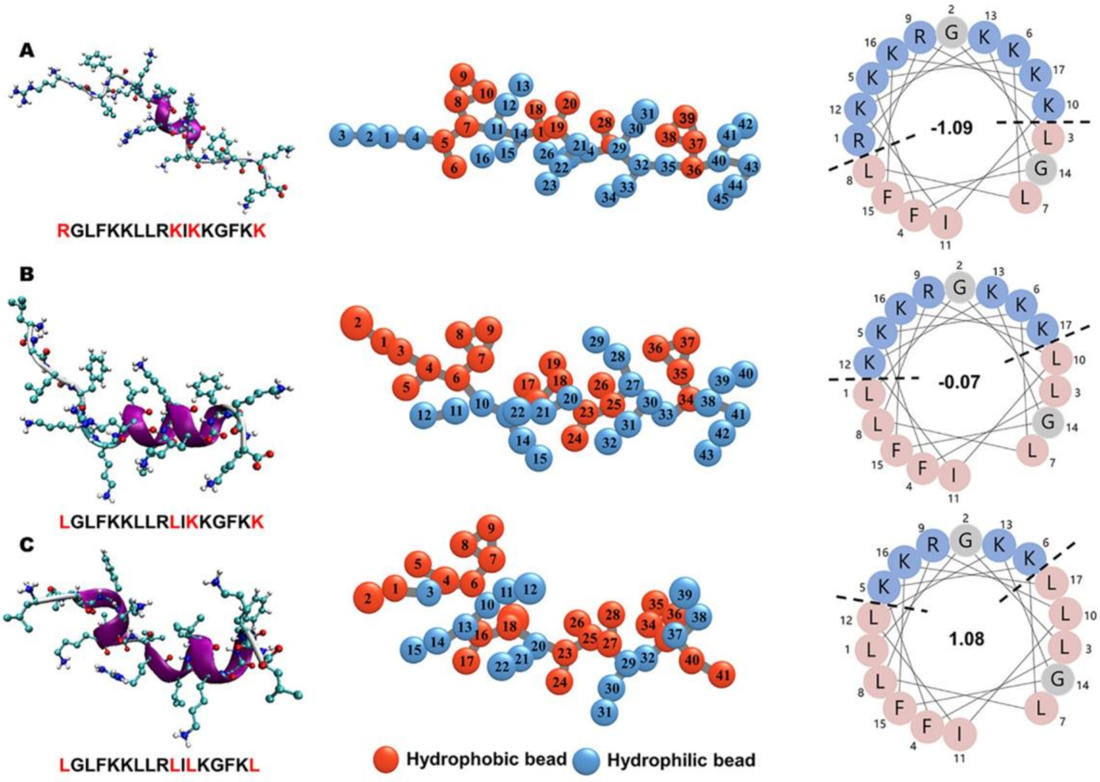

Figure S1. Schematic diagram of all-atom (left) and coarse-grained (middle) molecular structures as well as helical wheel representations (right) of (A) AS4-1, (B) AS4-5, and (C) AS4-9. In the coarse-grained models, hydrophilic residues are represented by blue beads and hydrophobic residues are represented by red beads. In the helical wheels, the hydrophilic residues are shown in blue and hydrophobic residues are shown in light pink. The sequence numbers corresponding to the residues and coarse-grained bead types are given in Table S1.

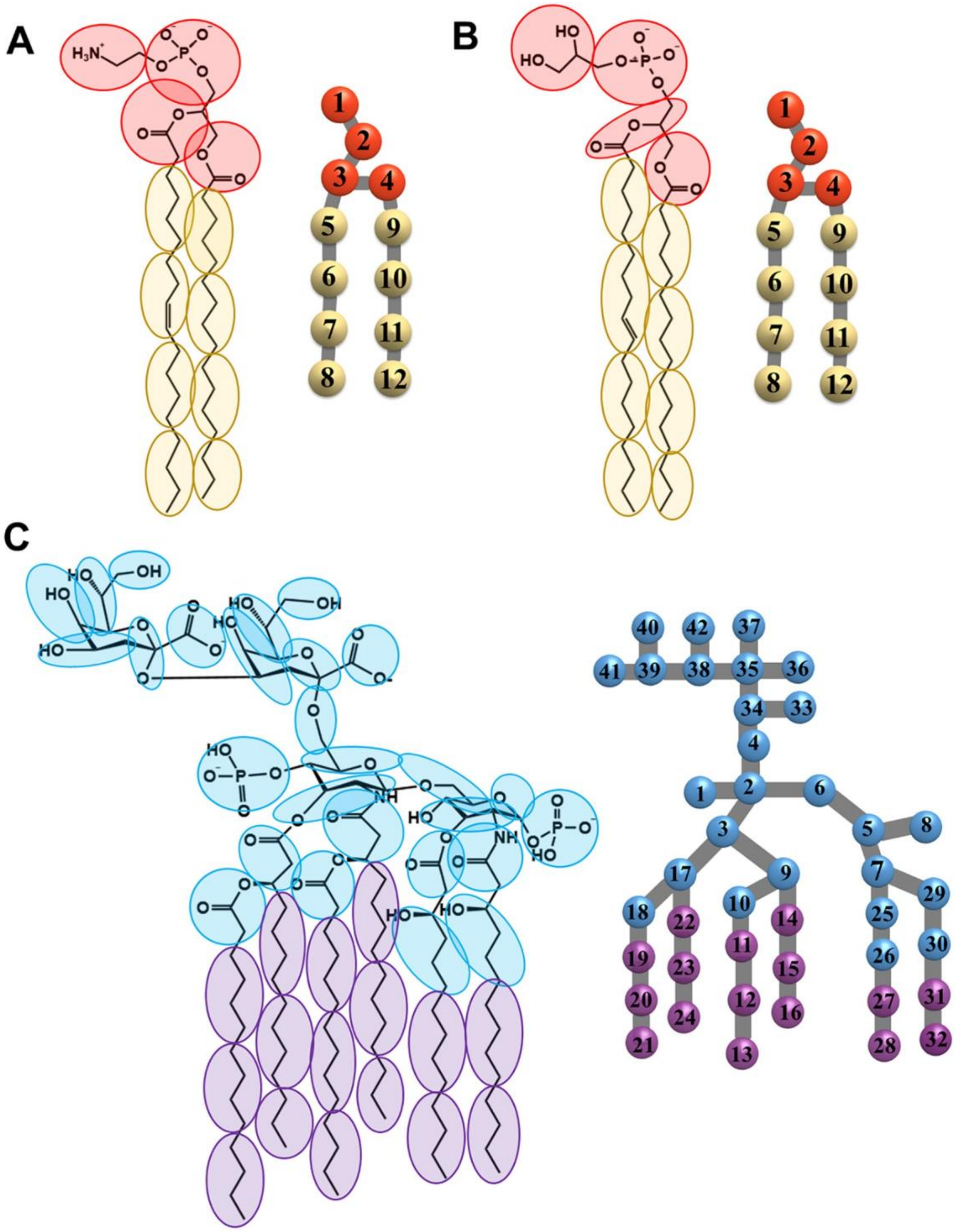

Figure S2. Molecular structures of POPE (A), POPG (B), and Re-LPS (C) and their corresponding coarse-grained mappings. For POPE and POPG, the coarse-grained hydrophilic head beads are in red; the hydrophobic tail beads are in yellow. For Re-LPS, the hydrophilic head beads are in cyan, and the hydrophobic tail beads are in purple.

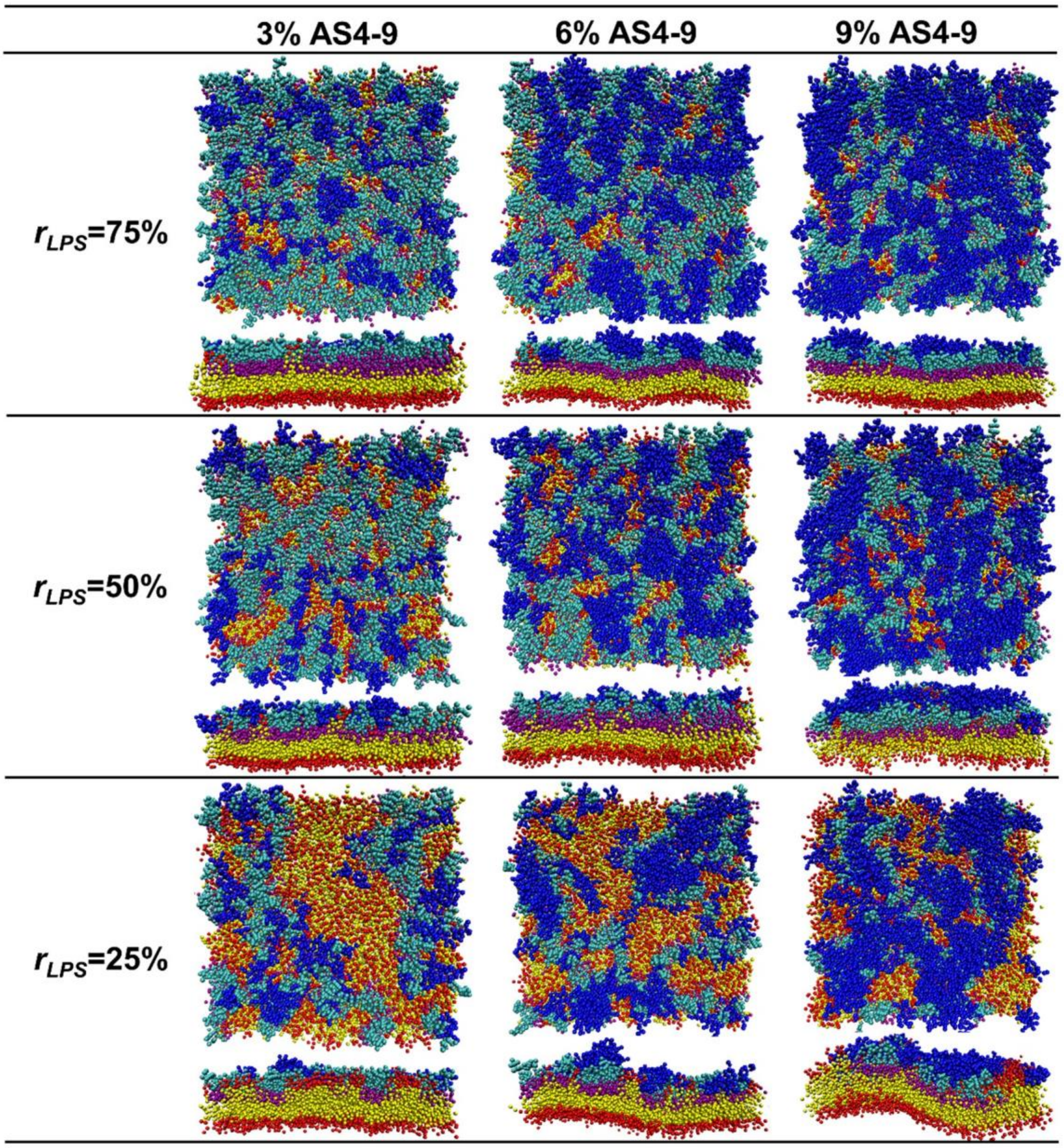

Figure S3. Top and side views of snapshots of AS4-9-treated scrambled OM with varying deficiencies of Re-LPS.

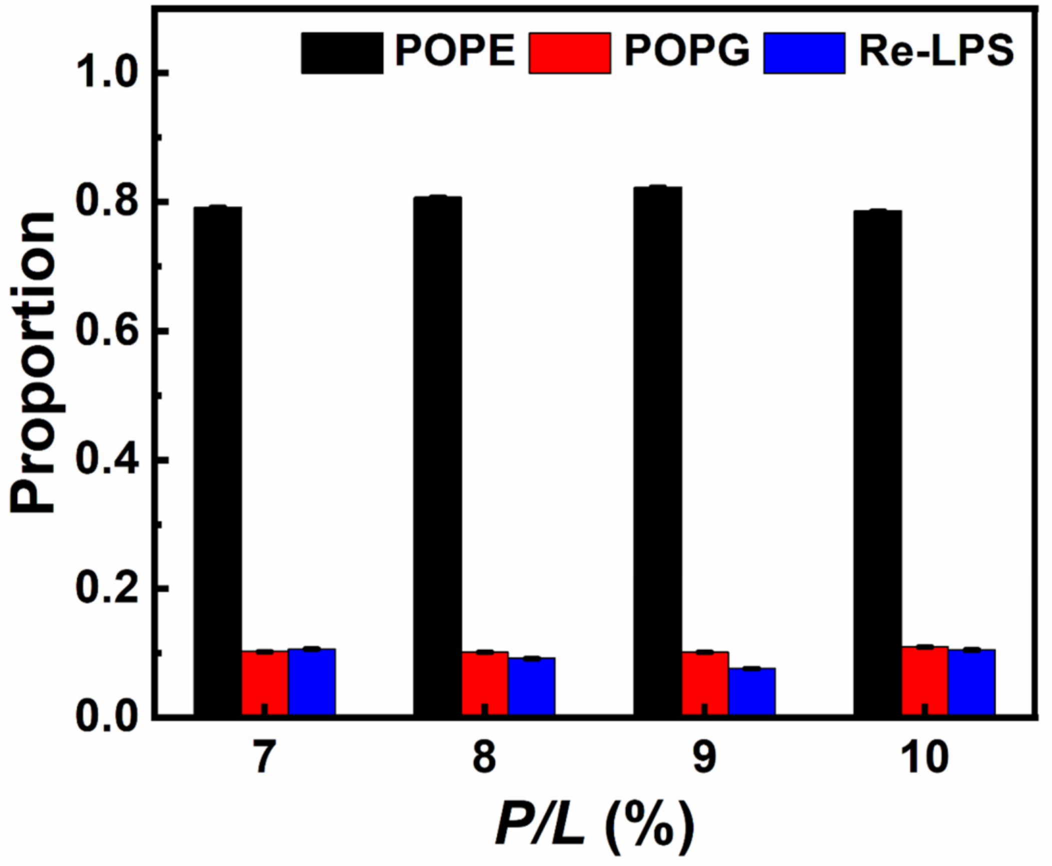

Figure S4. Proportion of different lipids in the buds of lipid-scrambled OM (*rLPS* = 10%) treated with AS4-9.

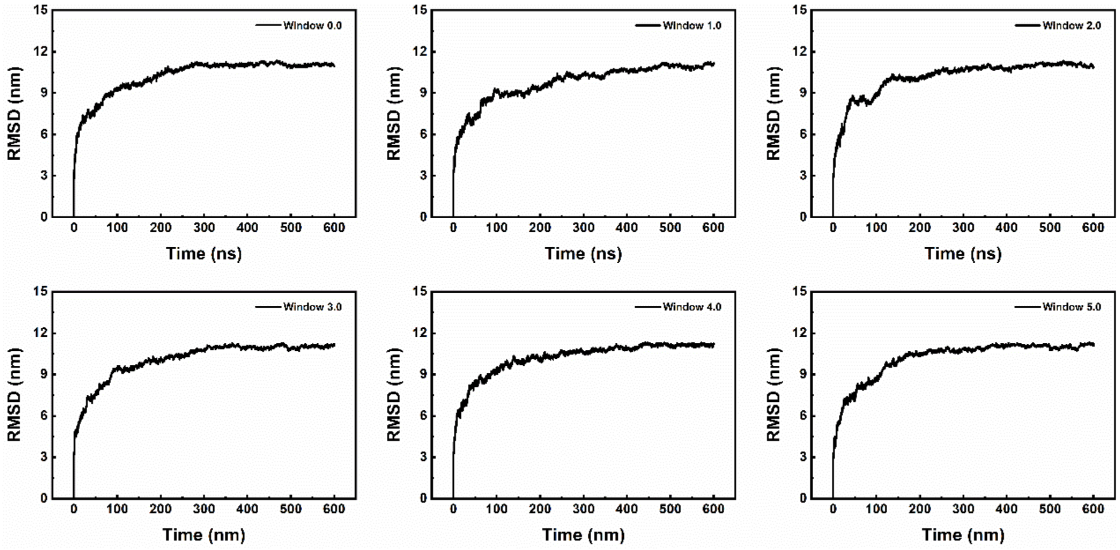

Figure S5. Root-mean-square-deviation (RMSD) of the OM in different windows for a POPE flipping from the phospholipid leaflet to the Re-LPS leaflet bound by AS4-9 at the peptide concentration of P/L = 3%.
